# Effective decellularisation of human saphenous veins for biocompatible arterial tissue engineering applications: Bench optimisation and feasibility in vivo testing

**DOI:** 10.1177/2041731420987529

**Published:** 2021-03-29

**Authors:** Nadiah S Sulaiman, Andrew R Bond, Vito D Bruno, John Joseph, Jason L Johnson, M-Saadeh Suleiman, Sarah J George, Raimondo Ascione

**Affiliations:** 1 Bristol Heart Insitute and Translational Biomedical Research Centre, Translational Health Sciences, Bristol Medical School, University of Bristol, Bristol Royal Infirmary, Bristol, UK; 2 Centre for Tissue Engineering and Regenerative Medicine, Universiti Kebangsaan Malaysia Medical Centre, Jalan Yaacob Latif, Cheras, Kuala Lumpur, Malaysia; 3 Centre for Nanosciences and Molecular Medicine, Amrita Institute of Medical Sciences and Research Centre, Amrita Vishwa Vidyapeetham, Kochi, India

**Keywords:** Decellularisation, bioengineering, tissue engineering, vascular graft

## Abstract

Human saphenous vein (hSV) and synthetic grafts are commonly used conduits in vascular grafting, despite high failure rates. Decellularising hSVs (D-hSVs) to produce vascular scaffolds might be an effective alternative. We assessed the effectiveness of a detergent-based method using 0% to 1% sodium dodecyl sulphate (SDS) to decellularise hSV. Decellularisation effectiveness was measured in vitro by nuclear counting, DNA content, residual cell viability, extracellular matrix integrity and mechanical strength. Cytotoxicity was assessed on human and porcine cells. The most effective SDS concentration was used to prepare D-hSV grafts that underwent preliminary in vivo testing using a porcine carotid artery replacement model. Effective decellularisation was achieved with 0.01% SDS, and D-hSVs were biocompatible after seeding. In vivo xeno-transplantation confirmed excellent mechanical strength and biocompatibility with recruitment of host cells without mechanical failure, and a 50% patency rate at 4-weeks. We have developed a simple biocompatible methodology to effectively decellularise hSVs. This could enhance vascular tissue engineering toward future clinical applications.

## Introduction

Coronary artery (CABG) and peripheral vascular bypass grafting (PVBG) are currently the most effective treatment to normalize blood supply to the ischemic myocardium or limbs following severe coronary or peripheral vascular atherosclerosis. To this end, various conduits are used including autologous vessels or synthetic grafts such as polytetrafluoroethylene or polyethylene terephthalate. The internal mammary artery (IMA) is the gold standard autologous conduit for CABG surgery improving long-term survival and life expectancy by ensuring a patency rate of 85% at 10 years.^1^ Yet, >70% of CABG grafts are based on the use of autologous greater saphenous vein (SVG), which, along with synthetic grafts, is the only available option for PVBG surgery.^2^ However, 15% to 20% of SVG grafts suffer early thrombosis at 1 year and 50% are occluded at 10 years due to thrombosis or intimal hyperplasia.^3^ A large proportion of synthetic and venous grafts used for PVBG get blocked at 2 to 4 years due to excessive thrombogenicity and complications from infection, with the patency steadily decreasing over time.^2^ The sub-optimal performance of SVG and synthetic grafts makes more effective conduits desperately needed.

Vascular tissue engineering involves biomaterials, cells and regulatory signals, and relies on optimisation of biomechanical cues and tissue architecture, including strategies for manufacturing small (<6 mm diameter) tissue engineered vascular grafts (TEVG).^4,5^ TEVGs have been developed using either ex vivo scaffolds seeded with cells and/or growth factors or biohybrid tissue with or without cell stripping (decellularisation). Decellularisation aims to remove the cellular components to prevent immune rejection or tissue mis-match at transplantation while preserving the extracellular matrix (ECM) and structural integrity. It is being used to bio-engineer skin, pericardium, heart valves and blood vessels^6–8^ with claimed advantages over synthetic materials being superior biocompatibility and reduced infections.^9,10^ However, concerns remain on the safety of decellularisation methods, cytotoxicity, biocompatibility as well as the clinical applicability in preclinical models highly relevant to humans.^11–13^ Effective decellularisation should aim for lack of residual viable cells and nuclear material, ECM integrity, <50 ng deoxyribonucleic acid (DNA) per mg dry weight ECM, and <200 base pairs of double stranded DNA (dsDNA) fragment length.^8,14^ Methods might differ based on type and size of tissue or organ to be decellularised.

It is suggested that key aspects of the ideal TEVG include biocompatibility, mechanical properties similar to arteries, readily available, low manufacturing costs, easy storage and be sterilisable.^15^

In this study, we used adapted mechanical and chemical methods described by others^16^ to effectively decellularise human SV (D-hSV) while keeping them biocompatible. Mechanical safety, biocompatibility, immunogenicity and surgical feasibility of the modified protocol were tested in vitro and in vivo using a porcine model of carotid artery replacement with no immunosuppression.

## Methods

### Human and porcine tissue collection

Segments of human SV left over from CABG surgery at the Bristol Heart Institute, Bristol, UK, were collected under sterile conditions and stored in either saline or Dulbecco’s modiﬁed Eagle's medium (DMEM) (Sigma) containing Penicillin-Streptomycin (200 μg/mL, Sigma); GlutaMAX™-I Supplement (2 mM, Sigma) stored at 4°C until decellularisation. Pre-decellularisation segments were retained (control) fixed in 10% formalin (Sigma) at 4°C for comparative histological analysis. Human tissue was obtained in accordance with ethical approval from the local committee (REC reference number: 10/H0107/63).

Fresh porcine carotid artery (pCA) were harvested under clinical conditions from control animals undergoing termination procedures (under Home Office license PPL: 30/2854) at the Translational Biomedical Research Centre (TBRC), Bristol, UK. These were placed in 50 mL Falcon tubes containing sterile DMEM supplemented as above and transported to the laboratory for processing to isolate endothelial (PCAEC) and smooth muscle (PCASMC) cells, as follows. After removal of surrounding adventitial tissue from pCA, vessels were cut longitudinally and PCAEC isolated by scraping the lumen with a scalpel. Cells were transferred to Endothelial Cell Growth Medium MV2 (Promocell), spun, resuspended and seeded on fibronectin-coated (100 µg/mL, Sigma) 6-well plates for culture (5% CO2 at 37°C). PCAECs were maintained until cells were 70-80% confluent before trypsinization for further analysis. PCASMC were cultured and expanded from 1 mm2 explants of the medial layer of vessels in Smooth Muscle Cell Growth Medium 2 (Promocell). Both cell types underwent standard culture procedures until use.

### SDS Cytotoxicity and optimisation of concentration

An initial stock solution of 1% SDS (w/v; Fisher Scientific) as highest concentration was made in sterile PBS, which equates to a 35 mM solution, and filtered through a 0.2 µm syringe filter. This stock was diluted further to achieve the decremental concentrations required (Supplemental Table 1). To ascertain the minimal non-cytotoxic concentration, SDS was added to fresh PCAECs and PCASMCs. The number of proliferating and viable cells was determined using EdU and AlamarBlue assay respectively, as described in the supplementary methods section.

### Optimisation of human saphenous vein decellularisation

To determine the optimum SDS concentration for successful decellularisation, hSV (*n* = 6) were cut into rings (0.5 cm length) placed in 5 mL increasing SDS concentrations (0.01; 0.025; 0.05; 0.075 and 0.1% (w/v in PBS)) and agitated on a tube roller (60 rpm, Stuart, UK) for 24 h at room temperature. SDS treated segments were transferred to PBS (Sigma) on the roller at room temperature with PBS changes after 24 h (Wash 1) and 48 h (Wash 2) before final storage in fresh sterile PBS at 4°C. To assess whether our protocol would translate to clinically relevant lengths of vein, we also undertook decellularisation of longer (4–6 cm) hSV segments. Obtained hSVs were divided into two pieces and placed either in 40 mL 0.01% SDS (w/v) on a tube roller (at 60 rpm) (D-hSV_ROLLER_) or cannulated and placed in a dynamic flow setup with 120 mL of 0.01% SDS (w/v) constantly recirculated for 24 h at room temperature through the vessel lumen, using a peristaltic pump at a flow rate of 6 mL/min (D-hSV_FLOW_). Next, SDS was replaced with PBS for two 24-h washes at room temperature. The efficacy of the decellularisation process was assessed as described for 0.5 cm segments with comparisons made versus long hSV controls and versus short D-hSVs.

### Evaluation of effectiveness of decellularisation

Formalin-fixed paraffin embedded transverse sections (FFPE; 5 µm thick) were stained with H&E to detect cell nuclei in the intima, media and adventitia by automated image analysis (ImageJ software) in four quadrants of four transverse sections (total of 16 counts). Counts were averaged for each untreated hSV control (*n* = 10) and each decellularised hSV segment (*n* = 10). The percentage of decellularisation was calculated compared to native control.

Double-stranded DNA content of tissue homogenates of native control versus decellularised hSVs (*n* = 10 per treatment) was assessed with a Quant-iT PicoGreen assay (Life Technologies) as described in the supplementary methods.

For long hSVs, to confirm that residual cell debris was non-proliferative, a BrdU cell proliferation assay was performed on D-hSVs on the roller with 0.01% SDS (w/v) (*n* = 5). Two cm segments of either native or D-hSV_ROLLER_ were cut open longitudinally to expose the whole lumen and pinned into a culture dish containing Sylgard resin. Opened D-hSV-roller were cultured for 14 days in RPMI 1640 containing 30% FCS, L-Glutamine, gentamicin, penicillin and streptomycin, and 10 µM BrdU (Sigma) (at 37°C, 5% CO_2_) with media changes every 2 to 3 days, before being fixed and paraffin embedded. Samples of native controls and D-hSVs were fixed at Day 0 to serve as control. Sections (5 µm thick) were probed with a mouse anti-BrdU antibody (Sigma, UK) and the percentage of BrdU positive cells calculated.

### Preservation of extracellular matrix integrity

hSVs before and after SDS treatment (*n* = 6 per treatment group) were compared with regard to content of hydroxyproline (Sigma), elastin (Fastin^TM^Elastin kit, Biocolor) and glycosaminoglycans (GAGs, Blyscan Assay Kit, Biocolor). Assays were performed according to manufacturer’s instructions on approximately 10 mg of tissue following treatment with 0, 0.01, 0.025, 0.025, 0.075 and 0.1% w/v SDS. All data were normalised to the total wet protein content of tissue, as determined by Pierce Detergent Compatible Bradford Assay (ThermoFisher, UK). In addition, FFPE transverse sections (5 µm thick) through the conduit were stained with picrosirius red (PSR), elastin van gieson (EVG) and alcian blue for evaluation of collagen, elastin and GAGs respectively. The percentage area of tissue staining for elastin, collagen and GAGs was determined by counting the number of pixels stained for each ECM component, within four quadrants in four transverse sections, and counted using automated image analysis software (ImageJ).

### Biocompatibility of decellularised hSVs

The viability of human adipose-derived stem cells (ADSC; Lonza), human umbilical vein endothelial cells (HUVEC; PromoCell), and human saphenous vein smooth muscle cells (HSVSMC; isolated using method described previously for PCASMC) seeded onto the D-hSVs was assessed with AlamarBlue (for details see supplementary methods).

To assess differences in biocompatibility between D-hSV_ROLLER_ and D-hSV_FLOW_ these were seeded with 5 × 10^4^ pCA ECs for 48 h, and then formalin-fixed before subsequently being stained with H&E, or immunostained with an endothelial cell marker (rabbit polyclonal to CD31 antibody; 0.9 µg/ml; Abcam, Ab28364) followed by a Alexa-Fluor 488 secondary antibody (chicken polyclonal anti-rabbit IgG; 10 µg/ml, ThermoFisher Scientific, A-21441), and a smooth muscle cell marker (mouse anti-actin, monoclonal 1A4, alpha smooth muscle-Cy3 antibody; 3 µg/ml; Sigma, C6198). Native hSV tissue was used as control.

### Quantification of residual SDS on decellularised hSVs

During the decellularisation process with 0.01% SDS, aliquots of every effluent (0.01% SDS, PBS Wash 1 and Wash 2) were collected after each step during both roller and flow protocols (*n* = 8 per protocol). The same hSVs (*n* = 5) were divided in two and subjected to either roller or flow protocols. Following decellularisation and washing steps, the concentration of SDS remaining was analysed using a methylene blue assay (see supplementary methods section).

### Mechanical strength testing before and after decellularisation

D-hSV in 0.01% SDS (w/v) (*n* = 5; D-hSV_ROLLER_), non-treated hSV control (*n* = 5), and porcine carotid artery (PCA; *n* = 5, as an arterial control) were stored in PBS (containing 0.02% Sodium Azide), before being mechanically tested at the ISO accredited testing facility (Amrita Centre for Nanosciences and Molecular Medicine, India). Burst pressure was calculated by inflating the conduits with water at a steady rate until the burst pressure was reached. The radial compliance was calculated by measuring the internal diameter change with a laser micrometer at pressures between 110 and 150 mmHg (conduits inflated with air); results are presented as percentage of diameter change per 100 mmHg.

### Assessment of mechanical strength, biocompatibility and patency rates in vivo

Following decellularisation on a roller with 0.01% SDS (w/v), D-hSV (2 cm length) were stored in sterile PBS at 4°C. In vivo transplantation was carried out at the advanced TBRC facility for large animals (as described in Supplementary methods), in female Landrace pigs (*n* = 6; mean weight 58.8 ± 1.4 kg), under strict clinical standards including anti-platelet therapy (first three pigs received 75 mg aspirin with food from 5 days before surgery till termination; the subsequent three pigs received 300 mg aspirin at the same time points) and anticoagulation with heparin to keep the activated clotting time > 250 s. Pigs were recovered and maintained under optimal animal welfare conditions for 4 weeks. After this period of maintenance, animals were subjected to general anaesthesia and mechanical ventilation. The implanted grafts were surgically exposed and evaluated. Next, they were excised, and formalin fixed, along with a section of the proximal and distal carotid artery and sectioned at 5 µm thickness. Sections were stained with H&E and EVG to analyse conduit lumen diameter and wall thickness (ImageJ Software).

## Statistics

All data is presented as mean ± SEM, and statistical analysis was performed using GraphPad Prism (GraphPad Software, California, USA), with differences between groups/treatments deemed significant if *p* < 0.05. The statistical test used for each comparison is shown in Supplementary Table 2.

## Results

### SDS Cytotoxicity and optimisation of concentration

Higher SDS concentrations reduced the proliferation and viability of PCAECs and PCASMCs (Table 1). PCAECs viability dropped below 70% at SDS concentrations ⩾0.00025%, although it was not until 0.005% that viability was significantly decreased (*p* < 0.01). At SDS concentrations <1.0 × 10^–4^% PCAECs proliferation was higher than the threshold viability (*p* < 0.05), but it dropped below 70% at 7.5 × 10^–4^%, with no proliferation at all observed at 2.5 × 10^–3^% (87 µM). PCASMCs tolerated higher SDS concentrations with proliferation and viability falling below 70% at ⩾7.5 × 10^–4^% and ⩾5.0 × 10^–4^, respectively.

**Table 1.** Viability and proliferation of PCAEC (*n* = 8) and PCASMC (*n* = 6) after 4 h exposure to different concentrations of SDS.

		SDS % w/v											
		0	0.000075	0.0001	0.00025	0.0005	0.00075	0.001	0.0025	0.005	0.0075	0.01	1
PCAEC (*n* = 8)	Viability (% of control)	100 ± 0.0	81.5 ± 9.6	72.9 ± 10.0	63.9 ± 8.7	65.1 ± 8.9	67.8 ± 8.9	63.6 ± 8.7	65.7 ± 8.9	52.6 **±** 3.2*	52.9 **±** 2.9*	51.8 **±** 2.8*	40.5 **±** 2.7*
	Proliferation (% of control)	100 ± 0.0	85.1 **±** 3.5*	83.5 **±** 3.0*	81.3 ± 3.7	72.3 ± 5.2	58.5 ± 9.9	37.2 ± 12.1	0.0 **±** 0.0*	0.0 **±** 0.0*	0.0 **±** 0.0*	0.0 **±** 0.0*	0.0 **±** 0.0*
PCASMC (*n* = 6)	Viability (% of control)	100 ± 0.0	85.3 ± 10.1	82.6 ± 10.5	73.0 ± 6.9	62.0 ± 4.4	63.1 ± 4.2	59.8 ± 2.7	60.4 ± 3.2	59.3 ± 3.1	59.7 ± 3.1	53.4 **±** 2.5*	39.4 **±** 4.0*
	Proliferation (% of control)	100 ± 0.0	93.6 **±** 1.3*	89.6 **±** 2.5*	84.5 **±** 2.9*	76.9 ± 4.0	68.9 ± 3.2	62.8 ± 3.9	37.9 **±** 7.6*	9.1 **±** 3.9*	0.0 **±** 0.0*	0.0 **±** 0.0*	0.0 **±** 0.0*

SDS: sodium dodecyl sulfate; PCAEC: porcine carotid artery endothelial cell; PCASMC: porcine carotid artery smooth muscle cell.

Values represent mean ± SEM.

* Indicates significant difference (*p* < 0.01) to 70% of control cellular viability and proliferation.

### Efficacy of decellularisation methods used

**Figure 1.**
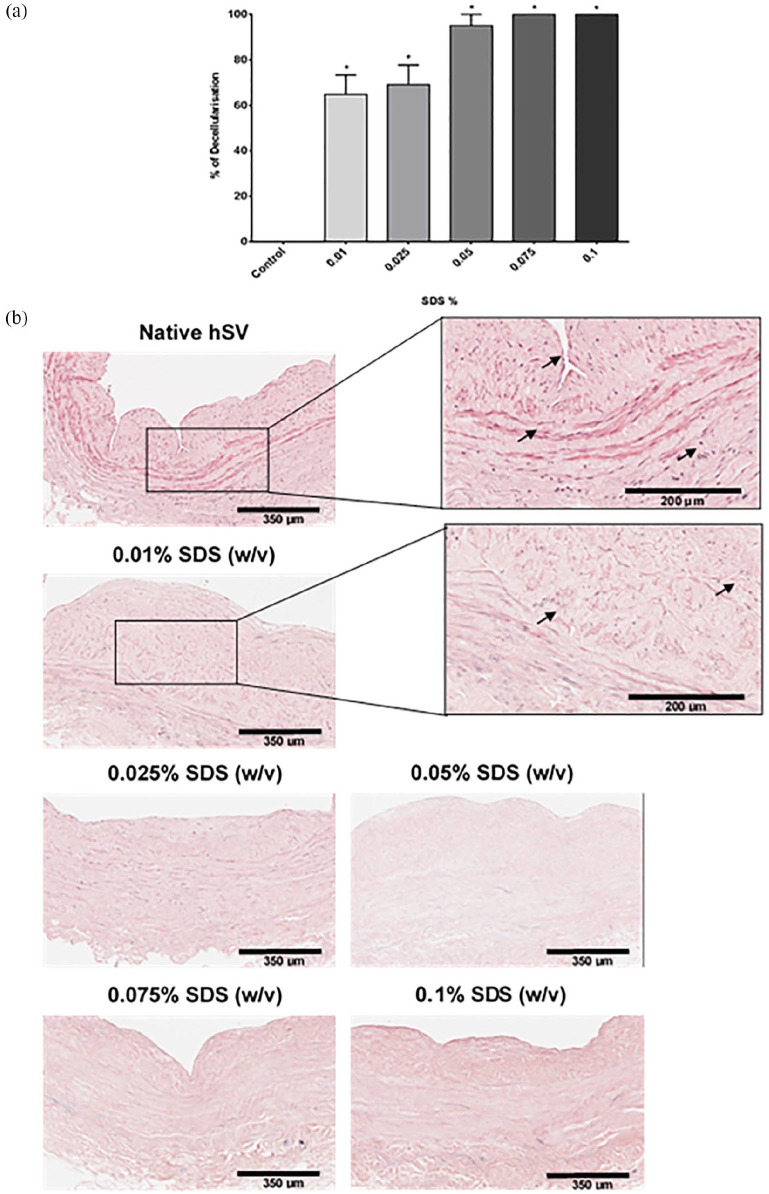
Efficiency of the decellularisation protocol with various SDS concentrations. Decellularisation protocol optimisation using 0.01, 0.025, 0.05, 0.075 and 0.1% SDS (w/v) compared to native control hSV. (a) Percentage of decellularisation was calculated by quantifying the nuclei (purple-black) in H&E stained FFPE sections of decellularised compared to native veins (*n* = 6). Values are presented as mean ± SEM, *indicates *p* < 0.001. (b) Representative images of decellularised and native veins stained with H&E. Scale bars represent 350 μm and 200 μm as marked.

**Figure 2.**
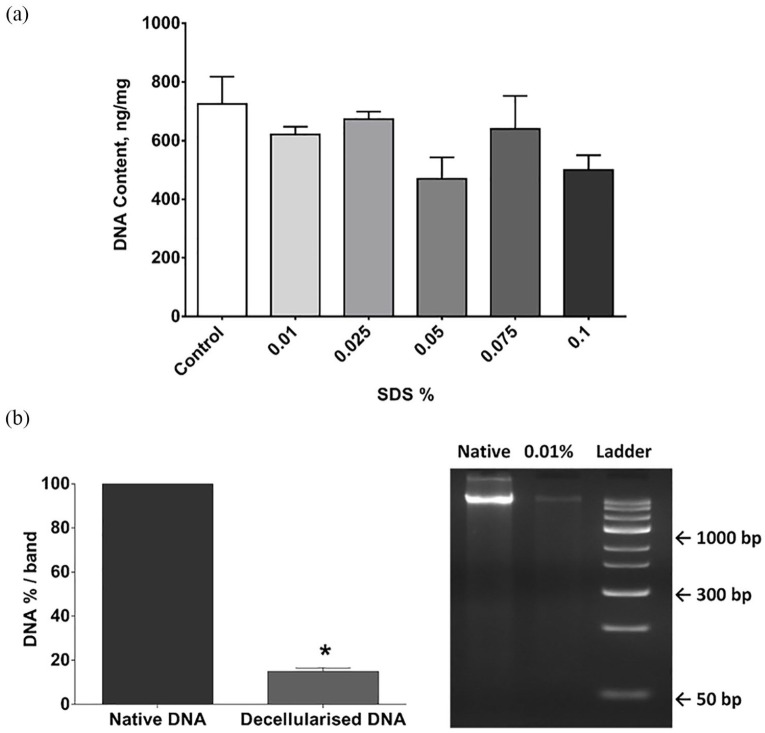
Effect of SDS treatment on DNA content. (a) PicoGreen assay to quantify dsDNA in native (control) hSV and following treatment in SDS concentrations of 0.01% to 0.1% (w/v) (*n* = 6). Values are presented as mean ± SEM. (b) Percentage of high molecular weight DNA in 0.01% decellularised and native tissues (*n* = 3) in agarose gel electrophoresis with a representative gel electrophoresis image of native hSV (Native) and 0.01% (w/v) SDS decellularised hSV. * indicates *p*<0.01 versus native DNA.

**Figure 3.**
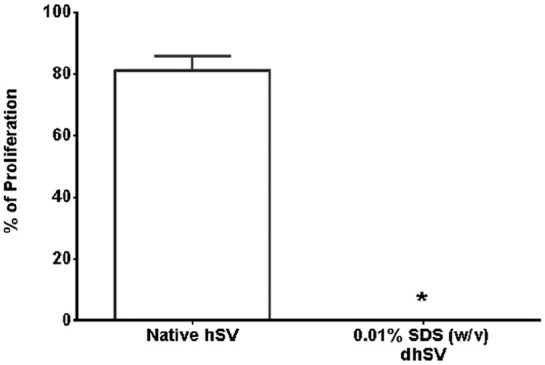
Detection of percentage of proliferating cells in organ cultures (*n* = 5), assessed by BrdU incorporation in native hSV and following treatment with 0.01% (w/v) SDS. *indicates *p* < 0.0001.

In short D-hSV segments (~0.5 cm) all SDS concentrations tested reduced the number of nuclei detected compared to controls (*p* < 0.001; Figure 1(a)), with the staining intensity of the residual nuclei being decreased (Figure 1(b)). While dsDNA content within the D-hSV did not differ from control for any of the SDS concentrations used (*p* > 0.05, *n* = 6; Figure 2(a)), most of the high molecular weight DNA had disappeared with 0.01% (w/v) SDS, when samples underwent agarose gel electrophoresis (*n* = 3; Figure 2(b)). No proliferating cells were detected by BrdU incorporation in D-hSVs when exposed to 0.01% SDS (the lowest concentration used) after 14 days in culture vs 81.0 ± 4.8% in controls (*p* < 0.0001; Figure 3).

**Figure 4.**
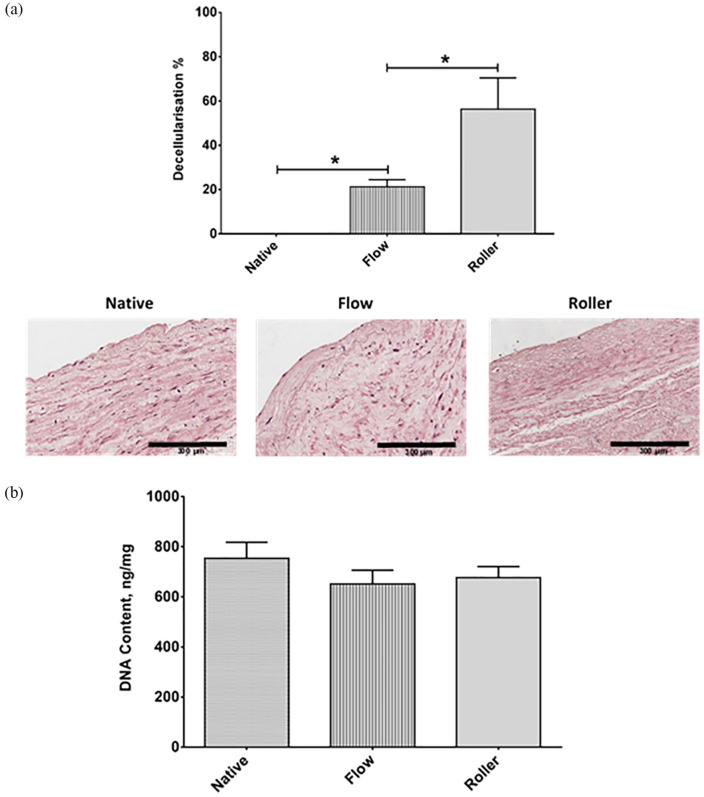
Effectiveness of decellularisation: flow versus roller protocols. (a) The percentage of decellularisation was calculated by quantifying the nuclei (blue) in H&E stained sections of decellularised and native veins. Representative images of native hSV and 0.01% SDS (w/v) flow versus roller decellularised hSVs are shown (*n* = 6). Scale bar represents 300 μm. (b) PicoGreen assay quantification of dsDNA from native hSVs and following decellularisation in 0.01% SDS (w/v) under flow or roller conditions (*n* = 10). Values are presented as mean ± SEM, *indicates *p* < 0.01.

As the results of 0.01% SDS (w/v) on short D-hSV were more encouraging than higher concentrations, we used next 0.01% SDS (w/v) to decellularise longer (4–6 cm) hSV segments to enhance translation. Long hSVs were exposed to 0.01% SDS on a roller and in a perfusion chamber setup. Decellularisation by flowing reduced the percentage of nuclei by 21.4 ± 3.1% versus controls (*p* < 0.05), whereas the rolling method reduced this by 56.3 ± 14.2%; *p* < 0.001 versus controls; Figure 4(a)). There was reduction of DNA content with both methods, but this did not reach significance (*p* > 0.05, Figure 4(b)).

**Figure 5.**
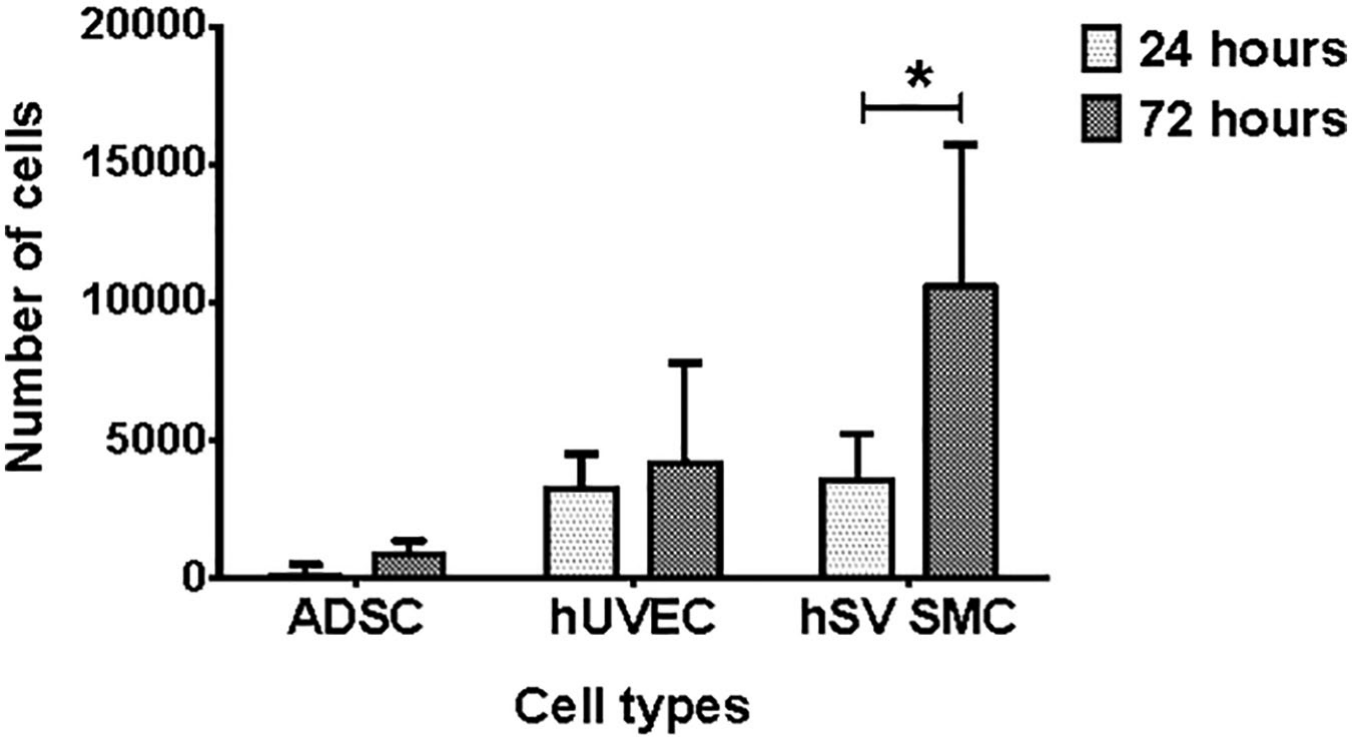
Cell viability of human adipose-derived stem cells (ADSC), umbilical vein endothelial cells (HUVEC) and saphenous vein smooth muscle cells (HSVSMCs) seeded onto hSV segments treated with SDS (0.01% w/v), and cultured for 24 and 72 h. The cell number was estimated with the Alamar Blue assay (*n* = 4). Values are presented as mean ± SEM, *indicates *p* < 0.05.

**Figure 6.**
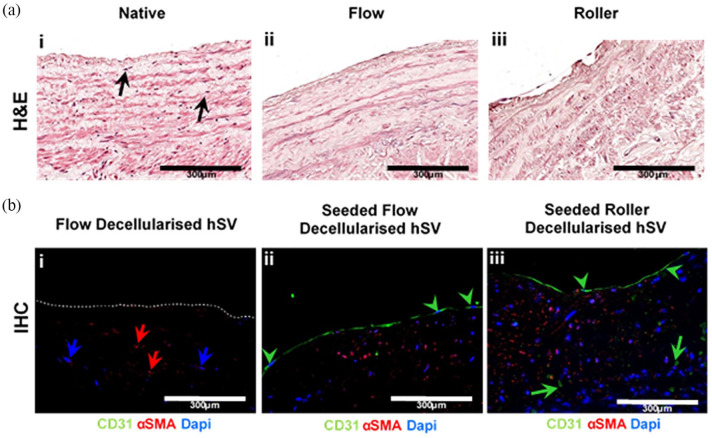
Assessment of biocompatibility using endothelial cell seeding and histological analyses. (a) Representative images of H&E stained sections of (i) native hSV, (ii) flow and (iii) roller decellularised hSVs that were seeded with pCAEC for 48 h. Black arrow showing nuclei. Scale bars represent 200 μm. (b) Representative images of dual immunohistochemistry for αSMA (red arrow) and CD31 (green arrow head and arrow) in (i) flow decellularised hSV; and the seeded (ii) flow and (iii) roller decellularised hSVs. Nuclei are stained with DAPI (blue arrow). Scale bars represent 300 μm.

For in vitro biocompatibility testing, opened D-hSVs-_ROLLER_ were seeded with ADSC, HUVEC and HSVSMC (*n* = 6 D-hSV per cell type). After 24 h, all cell types were viable, with ADSCs and HSVSMCs increasing their number over time (ADSC: 757 ± 741 to 3717 ± 1617 (*p* < 0.05); HUVEC: 2728 ± 707 to 2702 ± 1291 (*p* > 0.05); HSVSMC: 3559 ± 533 to 7836 ± 1587 (*p* < 0.05), 24–72 h, respectively) (Figure 5). Both D-hSV-_ROLLER_ and D-hSV_FLOW_ seeded with pCA-ECs showed positive H&E nuclei staining and positive staining for CD31 (Figure 6). In D-hSV_ROLLER_ H&E staining showed some nuclei within the tissue along with some positive staining for CD31 and αSMA.

### Preservation of ECM integrity

The analysis of tissue digests for short hSVs (~0.5 cm) showed that the hydroxyproline content of D-hSV was significantly decreased compared to untreated hSVs when exposed to SDS concentrations of 0.05% (w/v) and above (*p* < 0.05), whereas no significant changes were observed in elastin or GAG content. Pixel counting of stained D-hSV with PSR, EVG and alcian blue showed no significant reduction in collagen, elastin or GAGs content respectively, compared to untreated hSV (*p* > 0.05) (Table 2). In long D-SVs the content of hydroxyproline, elastin and GAG was not affected by 0.01% SDS (w/v) compared to controls for both D-hSV_ROLLER_ or D-hSV_FLOW_ protocols (*n* = 7, *p* > 0.05) (Table 3).

**Table 2.** Extracellular matrix composition of 0.5 cm long segments of hSV following decellularisation in increasing concentrations of SDS.

Assay^#^/Stain^$^	ECM component	Units		SDS % w/v				
			Native hSV	0.01	0.025	0.05	0.075	0.1
Hydroxyproline^#^	Hydroxyproline	ng/µg total protein	0.02 ± 0.002	0.013 ± 0.001	0.014 ± 0.002	0.01 **±** 0.001*	0.01 **±** 0.002*	0.01 **±** 0.001**
PSR^$^	Collagen	% pixels/area	63.98 ± 5.52	67.13 ± 7.20	65.44 ± 4.88	54.00 ± 4.07	52.84 ± 2.69	49.00 ± 3.89
FastinElastin^#^	Elastin	ng/µg total protein	3.93 ± 0.67	3.59 ± 0.27	2.55 ± 0.17	2.74 ± 0.22	2.81 ± 0.26	2.73 ± 0.26
EVG^$^	Elastin	% pixels/area	32.05 ± 10.95	15.90 ± 2.97	20.52 ± 9.18	27.76 ± 8.95	18.62 ± 6.89	12.84 ± 1.58
Blyscan^#^	GAGs	ng/µg total protein	0.5 ± 0.05	0.4 ± 0.02	0.4 ± 0.02	0.38 ± 0.05	0.39 ± 0.03	0.4 ± 0.02
Alcian Blue^$^	GAGs	% pixels/area	80.25 ± 4.26	80.75 ± 3.04	82.91 ± 1.34	77.96 ± 3.90	74.74 ± 6.18	65.62 ± 7.80

ECM: extracellular matrix; SDS: sodium dodecyl sulfate; PSR: picrosirius red; EVG: elastin van gieson; GAGs: glycosaminoglycans; hSV: human saphenous vein.

Values represent mean ± SEM (*n* = 6).

* *p* < 0.05. **p < 0.01 compared with native hSV.

# Denotes assay used.

$ Denotes stain used for immunohistochemistry.

**Table 3.** Extracellular matrix composition of hSV tissue lysates following 0.01% SDS (w/v) decellularisation with flow and roller protocols.

ECM component	Units	Native hSV	DhSV_FLOW_	DhSV_ROLLER_
Hydroxyproline	ng/µg total protein	0.027 ± 0.003	0.021 ± 0.002	0.022 ± 0.003
Elastin		3.733 ± 0.158	3.319 ± 0.171	3.486 ± 0.176
GAGs		0.594 ± 0.063	0.410 ± 0.020	0.398 ± 0.041

ECM: extracellular matrix; GAGs: glycosaminoglycans; hSV: human saphenous vein; DhSV: decellularised hSV.

Values represent mean ± SEM (*n* = 7).

### Mechanical testing

**Figure 7.**
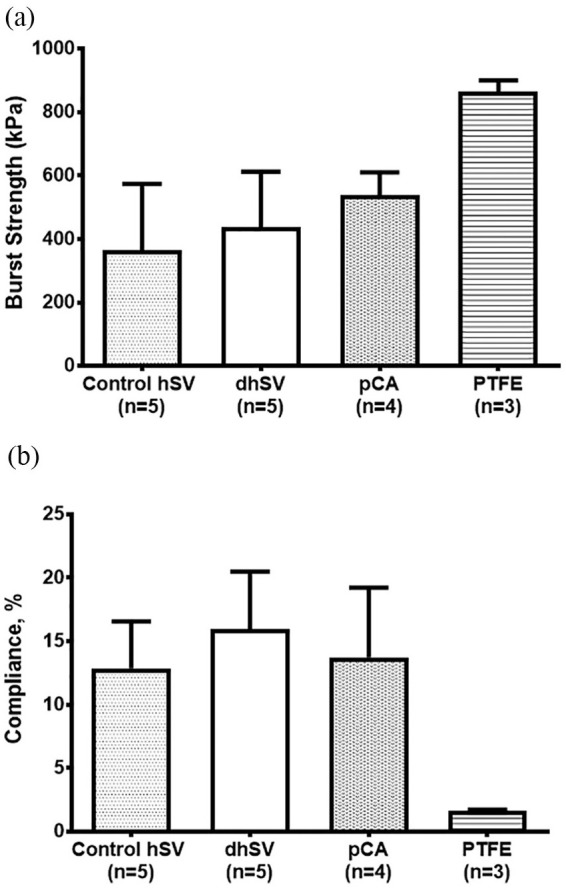
Burst strength (a) and compliance (b) of control (native) hSV, 0.01% SDS w/v decellularised hSV and porcine carotid artery (pCA) and PTFE segments. Values are presented as mean ± SEM. Number of samples indicated in graphs.

The burst strength of D-hSV treated with 0.01% SDS (436 ± 176.3 kPa; *n* = 5) was not significantly different from either untreated hSV controls (364 ± 209.4 kPa; *p* > 0.05, *n* = 5), untreated porcine carotid artery (537 ± 73 kPa; *p* > 0.05, *n* = 4) or synthetic PTFE (863.7 ± 36.2 kPa, *p* > 0.05, *n* = 3) (Figure 7(a)). The compliance of native hSV (12.9 ± 3.7%/100 mmHg, *n* = 5), PCA (13.7 ± 5.5%/100 mmHg, *n* = 4) and synthetic PTFE (1.6 ± 0.11 5%/100 mmHg, *p* > 0.05, *n* = 3) were also not significantly different from the D-hSV (D-hSV: 16.0 ± 4.5 %/100 mmHg, *p* > 0.05, *n* = 5) (Figure 7(b)).

### Residual SDS concentration

**Figure 8.**
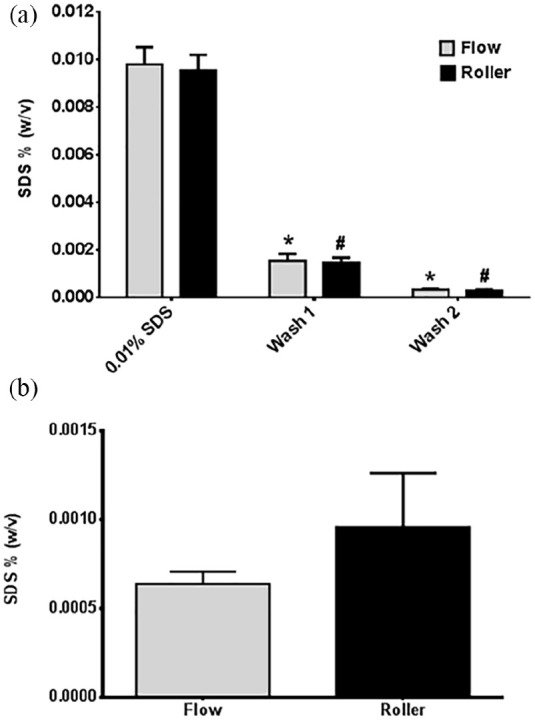
Effectiveness of the removal of SDS in the flow compared to roller decellularisation protocols. (a) The concentration of SDS in the effluent for both protocols after decellularisation with 0.01% (w/v) SDS (*n* = 8 per protocol) and (b) the residual amount per gram of tissue, in saphenous vein (*n* = 5 per protocol) at the end of the decellularisation protocol was determined using the methylene blue assay. Values are presented as mean ± SEM. * and # indicate significant difference (*p* < 0.001) compared to 0.01% SDS for flow and roller respectively.

Methylene Blue analysis of the effluent from D-hSV_FLOW_ and D-hSV_ROLLER_ (Figure 8(a)) showed that SDS was still detected in the first (D-hSV_FLOW_: 0.0016 ± 0.0003%; D-hSV_ROLLER_: 0.0015 ± 0.0002% D-hSV_ROLLER_) and second (D-hSV_FLOW_: 0.0003 ± 0.0000%; D-hSV_ROLLER_: 0.0003 ± 0.0000% D-hSV_ROLLER_) PBS washes, but these were both significantly reduced compared to the SDS effluent (D-hSV_FLOW_: 0.0098 ± 0.0007%; D-hSV_ROLLER_: 0.0095 ± 0.0006%, *p* < 0.001). Traces of SDS were detected in tissue decellularised by both methods (D-hSV_FLOW_, 0.0006 ± 0.0001%/g, *n* = 5; D-hSV_ROLLER_, 0.0010 ± 0.0003%/g, *n* = 5) but there were no differences between method (*p* > 0.05) (Figure 8(b)).

### Evaluation of D-hSVs following in vivo transplantation in pig

There was no obvious mismatch in lumen diameter and wall thickness between D-hSV and native carotid artery at surgical transplantation in 5/6 experiments. In experiment 5, the segment of D-hSV used was moderately dilated resulting in a moderate mismatch with the PCA within which it was sutured. The suturability of all grafts was excellent with easy passing through of 7-0 prolene stitches and no bleeding from the suture line following arterial reperfusion at systemic arterial pressure and normal pulsatility across the graft and distal to it. All six pigs survived the graft procedure and were extubated within 30 to 45 min from its completion. Animals gained normal weight during the following 4 weeks (mean preoperative weights 58.8 ± 1.4 kg vs. 76.3 ± 2.7 kg at explant procedure). Three out of six grafts were patent at 4-week post-surgery (50%). Of note, 1/3 grafts of the 75 mg aspirin cohort were patent (33%) compared to 2/3 grafts of the 300 mg aspiring cohort (66%).

Measurement of FFPE sections (Table 4) showed that there was no significant vessel inner diameter mismatch between D-hSV and native porcine carotid artery prior to implant, however the carotid artery had a 1.43 ± 0.12-fold thicker vessel wall (*p* < 0.05). At 4-weeks post-surgery, there was no overall change in inner diameter, however the wall thickness had increased in all patent grafts when compared to pre-implanted D-hSV (5.92 ± 0.66 -fold change, p < 0.05). The thickness of the graft wall was also thicker than that of the native pCA proximal and distal to the D-hSV graft (*p* < 0.05) (the exception being Pig 1, which had a thinner graft wall compared to the native distal pCA).

**Table 4.** Morphological analysis of vessels taken at time of graft implant, or at 4 weeks post-implant (*n* = 6).

	Conduit	Inner diameter (µm)	Wall thickness (µm)
Pre-implant	Native hSV	1875 ± 207	622 ± 31
	DhSV	2068 ± 193	463 ± 30
	Native PCA	2412 ± 339	658 ± 56*
Explanted tissue	DhSV Graft	2987 ± 1294	2802 ± 421*
	PCA Proximal	2457 ± 607	864 ± 142^†^
	PCA Distal	1371 ± 443	974 ± 175^†^

PCA: porcine carotid artery; hSV: human saphenous vein; DhSV: decellularised hSV.

Measurements were carried out on images of EVG stained formalin-fixed paraffin-embedded sections (*n* = 3).

* *p* < 0.05 versus pre-implant DhSV.

† *p* < 0.05 versus explanted DhSV Graft.

## Discussion

We describe the development and validation of a simple, rapid and biocompatible method for decellularising hSV for possible future use in coronary and/or vascular surgery. We utilise low SDS concentration and show that this achieves an effective hSV decellularisation with no residual viable cells (only DNA traces) while preserving the ECM composition and the mechanical strength, with excellent biocompatibility in vitro and in vivo when using an advanced porcine model of carotid artery replacement, without immunosuppression.

SDS contains a negatively charged functional group making it an anionic surfactant that acts on cell membranes thus disrupting their stability. It is routinely used in molecular biology as a cell lysis buffer as it also binds to, and eventually denatures, proteins by unfolding the protein 3-dimensional formation. Due to this mechanism of action on cells and their proteins, it is vital to optimise the exposure time and concentration of SDS used to find the right balance between cell removal and maintenance of the structural integrity of the decellularised vascular scaffold.

Using SDS 0.01% w/v, we achieved 65% decellularisation in short hSVs (~0.5 cm), with almost full decellularisation at 0.075% SDS. Schaner et al.,^16^ also observed almost complete cell loss at 0.075% SDS, however at 0.01% SDS they only achieved 32% cell loss. They carried out the decellularisation at 37°C in a shaking waterbath for 15 h, whereas we used room temperature on a roller for 24 h. Despite D-hSVs not being completely free of cell nuclei, H&E staining showed that residual nuclei were much fainter than in controls, implying reduced DNA content. Noticeably, there was no BrdU uptake by residual cells within the D-hSVs after 14-days of cell culture, compared to 81% of cells in untreated vein, hence demonstrating no cell viability within the D-hSV after treatment.

To ascertain the biocompatibility and translational potential of our approach we compared the H&E findings of D-hSV at 0.01% SDS with two commercially available decellularised biological surgical patches routinely used in clinical practice (CorMatrix^®^ ECM^®^ (CorMatrix Cardiovascular, Inc., USA) and No-React^®^ Porcine Pericardial Patch (Biointegral Surgical Inc., Canada)). This showed that the commercially available patches contained noticeably more nuclei compared to our D-hSV as a confirmation of the effectiveness of our approach and that residual nuclei do not preclude translation/clinical application.

The content of double-stranded DNA was not affected by any of the SDS concentrations tested. Conversely, we observed a decrease in fragments of high molecular weight DNA at 0.01% SDS. This discrepancy may be explained by lab methods used as small <50 bp fragments can be detected by the PicoGreen assay, but not by gel electrophoresis. It is suggested that the minimum criterion for successfully denatured DNA in decellularised tissue is <200 base pairs.^8^ Fragments <300 bp are reported to be less likely to induce inflammatory and tissue remodelling, with DNA fragments of this size found in commercially available scaffolds.^17^ Other studies have shown only 10% of DNA remains after SDS decellularisation of porcine myocardium, heart valve, small intestine submucosa and lung.^18–20^ However, these studies used much higher concentrations of SDS (0.1%–1% w/v). We could have added DNAses in our method to break down the DNA within hSV as done by others for different tissues,^21^ however DNAses have been shown to reduce mechanical stability and reduce GAG content of tissue, compared to methods incorporating DNAses.^21^ In addition, we believe this approach could have impacted negatively on the biocompatibility of D-hSVs, as incomplete removal of DNAses would have affected the cell seeding/proliferation, and longer wash steps would have been required for complete removal, potentially making the method less attractive translationally.

The preservation of the integrity of the ECM was evaluated using both histological staining for collagen, elastin and GAGs, alongside assays for hydroxyproline (as a major component of collagen), elastin, and GAGs. The only significant change in ECM integrity we observed was a decrease in hydroxyproline content at 0.05% SDS concentration. This finding was not seen with collagen staining, however differences in sensitivity between the assay and histological staining could account for this. Also, staining was carried out on 5 µm thick tissue sections, compared to approximately 100 µm thick pieces of tissue used in the assay. There was also a trend for decrease in elastin content at 0.025% SDS, but this was not significant.

We reasoned that 0.5 cm segments of hSV have very little potential for clinical applications and therefore thought to validate these findings on longer hSV segments (4–6 cm) using the 0.01% SDS concentration and expose them to roller agitation and flow perfusion settings, as perfusion methods are commonly used to decellularise heart, lung, liver and kidneys.^22–24^ We observed that the amount of decellularisation obtained was greater with the roller than with the flow apparatus, while DNA content did not differ in line with what observed for the short hSVs. Similarly, the perfusion method did not disrupt the ECM as levels of hydroxyproline, elastin or GAGs did not change and were comparable to those observed with the roller. However, it should be mentioned that we used a low flow system. High flow systems, possibly pulsatile, might have been more effective particularly when thinking that CABG surgery require SV of at least 12 to 15 cm^25,26^ that might be better decellularised by using high flow systems.

For the evaluation of residual mechanical strength of D-hSVs we used the burst strength and circumferential compliance. These methods are important indicators of graft suitability before proceeding to ex vivo flow assessment, or in vivo arterial models. In both cases, we found no significant differences between D-hSVs, untreated hSV, or untreated PCA. It has been shown that hSV and IMA have average burst pressures of 2134 mmHg (≡285 kPa) and 3073 mmHg (≡410 kPa) respectively, with the compliance of the vessels being 11.5%/100 mmHg (IMA) and 25.6%/100 mmHg (hSV).^4^ In the present study, D-hSVs had a higher burst pressure (436 kPa) and only 8.23%/100 mmHg compliance than untreated hSV controls. This confirmed the suitability of the D-hSVs as grafts for ex vivo and in vivo evaluations. In our case, the burst pressure (537 kPa) and compliance (13.7 mmHg) of the pig carotid artery were not significantly different to that of D-hSV supporting the safety of an in vivo implant in pig and as a good base for arterial tissue engineering.

The level of cytotoxicity of SDS varies between cell types with the threshold being 0.002% for human bronchial epithelial cells, lung fibroblasts and mesenchymal cells and 0.00012% for human pulmonary vascular endothelial cells.^27^ Mathapati et al.^28^ suggested that 0.1% SDS equates to a residual content of only 0.8 mg/g of implanted SV. In this study, we observed residual SDS of 0.0006%/g and 0.0010%/g of tissue for flow versus roller methods respectively. This equates to 0.6 mg/g and 1.0 mg/g respectively. These trace of SDS did not have a significant cytotoxic effect in vitro when these D-hSVs were seeded with porcine endothelial cells and smooth muscle cells in culture, or when their biocompatibility was assessed by seeding with human ADSC, HUVEC and HSVSMC. When added directly to porcine cells in culture, concentrations greater than 0.00025% SDS (PCAEC) and 0.0005% SDS (PCASMC) had a detrimental effect on cell viability, which fell below the ISO standard threshold of 70% of control cells. The proliferative capacity of cells was impaired at 0.00075% SDS for both PCAEC and PCASMC. On balancing viability and proliferation rates, endothelial cells appeared more susceptible to SDS cytoxicity at low concentration than SMCs. However, this may not be an issue as endothelial cells lining the lumen are in contact with D-hSVs only on the basal cell surface. With regard to methods of decellularisation used, the SDS levels found in D-hSV_FLOW_ should not affect cellular proliferation, whereas those found with D-hSV_ROLLER_ may reduce it. All the cell types seeded were viable, and among them HSVSMC significantly proliferated. HUVEC did not proliferate beyond the 48 h time-period, but this is to be expected, as once endothelial cells come into contact with each other and form a monolayer they exit the cell cycle and do not grow or proliferate further.^29^ PCAEC survived on D-hSVs regardless of the decellularisation protocol used with some CD31 positive cells observed within the wall of D-hSVs_ROLLER_, along some positive SMC actin staining observed in absence of SMCs seeding, which warrant further investigation.

We undertook xenotransplantation of D-hSVs in pigs with no immunosuppression. In the first cohort of three pigs we used 75 mg of daily aspirin with food. This was associated with a patency rate at 4 weeks of 33% (2/3 grafts fully thrombosed). In the second cohort of three experiments we used 300 mg of aspirin daily with food, also in line with clinical recommendations.^30^ In this second cohort the graft patency rate was 66% at 4 weeks (1/3 graft thrombosed), giving an overall patency rate of 50%, with a significant infiltration of the D-hSV grafts by host porcine cells. There was no mechanical failure of any of the grafts implanted and excellent suturability with no bleeding from the suture lines.

In conclusion, we have developed a simple and rapid methodology to effectively decellularise hSVs. This approach allows to obtain a tissue engineered acellular vascular scaffold with excellent biocompatibility and mechanical strength to enhance safe use for autologous or allogeneic clinical applications. We identify a low SDS concentration that achieves an effective balance between hSV decellularisation with no residual viable cells while preserving the integrity of the ECM composition. Preliminary in vivo feasibility testing in pig, in terms of mechanical safety and patency rates showed remarkable results when considering that these were xeno-transplants with no immunosuppression used. The approach used here has great potential both as a pre-clinical graft testing model, and in the clinical setting, as even during xenotransplantation without immunosuppression, the results are extremely positive. Autologous veins or those from a deceased donor could be harvested, and the resulting decellularised hSV may then undergo sophisticated arterial tissue engineering prior to surgical implantation for example, graft molecular functionalisation to harness host cells to ehance additional in-situ arterial tissue engineering.

## Supplemental Material

sj-docx-1-tej-10.1177_2041731420987529 – Supplemental material for Effective decellularisation of human saphenous veins for biocompatible arterial tissue engineering applications: Bench optimisation and feasibility in vivo testing Click here for additional data file. Supplemental material, sj-docx-1-tej-10.1177_2041731420987529 for Effective decellularisation of human saphenous veins for biocompatible arterial tissue engineering applications: Bench optimisation and feasibility in vivo testing by Nadiah S Sulaiman, Andrew R Bond, Vito D Bruno, John Joseph, Jason L Johnson, M-Saadeh Suleiman, Sarah J George and Raimondo Ascione in Journal of Tissue Engineering
