# Supplementary material for: Effective decellularisation of human saphenous veins for biocompatible arterial tissue engineering applications: Bench optimisation and feasibility in vivo testing
Source: J Tissue Eng. 2021 Mar 29;12:2041731420987529. doi: 10.1177/2041731420987529 (PMC8010838; doi:10.1177/2041731420987529)
Supplement: sj-docx-1-tej-10.1177_2041731420987529 – Supplemental material for Effective decellularisation of human saphenous veins for biocompatible arterial tissue engineering applications: Bench optimisation and feasibility in vivo testing [file sj-docx-1-tej-10.1177_2041731420987529.docx]

**Supplementary**

**Methods**

*SDS Cytotoxicity and optimisation of concentration*

Porcine carotid artery endothelial cells (PCAECs) (n=8 wells per concentration) and porcine carotid artery smooth muscle cells PCASMCs (n=6 wells per concentration) were seeded in a 96-well plate at a density of 1x10^5^ cells/cm^2^ and allowed to grow overnight. Cells were then rendered quiescent in media containing 2% FBS for 24 hours. SDS (Fisher Scientific, UK) (7.5x10^-5^ to 1%, w/v; equates to 2.6 µM to 35 mM; Suppl. Table 1) diluted in media containing 10μM 5-ethynyl-2'-deoxyuridine (EdU; Click-iT EdU Alexa Fluor 555 Imaging Kit, Life Technologies) was added to the cells for 24 hours, before fixation in 3% paraformaldehyde. EdU detection was performed according to manufacturer’s instructions. The number of proliferating cells were counted per microscope field (200x magnification). Five images of each well were captured, and nuclei counted using ImageJ software. The percentage of proliferating cells was calculated as the percentage of cells grown in normal media, which served as a positive control. The International Organisation for Standardization (ISO) has stated that the cytotoxic threshold for reagents compared to normal cell culture medium, be set at less than 70% critical viability^1^, so for statistical analysis our data were compared to 70% of the value obtained for untreated control cells.

AlamarBlue assay (Qiagen) was used to assess the effect of 4 hours of exposure to SDS on PCAECs and PCASMCs viability at a range of dilutions (7.5x10^-5^ to 1% as above).

Cell media was supplemented with 10% (v/v) AlamarBlue. On completion, the absorbance values were read on a microtiter plate reader at 570nm and 595nm while cell viability was calculated following manufacturers protocols. Data were compared to 70% critical viability as previously described.

*Evaluation of effectiveness of decellularisation*

DNA was extracted from native control versus D-hSVs using a salting-out method. Briefly, tissue samples (10-25mg) were lysed in TNES buffer containing Proteinase K, NaCl was added to the lysed samples, and the DNA precipitated out of solution using 100% ethanol, before being re-suspended in Tris-EDTA, pH 7.5, for use in the Quant-iT PicoGreen assay (Life Technologies) assay following manufacturer’s instructions.

*Biocompatibility of decellularised hSVs*

A biopsy punch was used to obtain 4mm discs of DhSV_ROLLER_ (n=6) subjected to 0.01% SDS, and these were mounted luminal surface upwards in CellCrowns (Sigma). Following pre-conditioning overnight in cell-type specific media, cells were seeded at a density of 5x10^4^ cells, and cultured for 24 hours. After 24 hours the media was discarded, and fresh media supplemented with 10% (v/v) AlamarBlue (Qiagen) added for 4 hours. The same process was repeated after 72 hours. An aliquot of AlamarBlue supplemented media was removed and absorbance values read on microtiter plate reader at 570nm and 595nm. Cell-type specific media without AlamarBlue was used to obtain baseline values. A standard curve was generated based on the AlamarBlue absorption for known numbers of cells, enabling calculation of the total number of viable cells present in test samples.

*Quantification of residual SDS on decellularised hSVs*

500mg of tissue was weighed and homogenised in liquid nitrogen; 75% ethanol was added prior to centrifugation at 21,000 rcf, with the resulting supernatant being aspirated and analysed using a modified two-phase methylene blue titration method ^2, 3^. 50 µl of methylene blue (0.0125% w/v; Sigma) was added to 50 µl of sample or SDS standards (0-0.01% w/v) and tubes vortexed vigorously. 200 µl of chloroform was added and tubes vortexed and incubated (30 mins, room temperature). A two-phase layer was observed and 150 µl of the bottom layer was transferred into a 96-well polypropylene plate, and immediately read at 630nm using a microtiter plate reader. Absorbance values were converted to SDS concentrations using a standard curve.

*Assessment of mechanical strength, biocompatibility and patency rates in vivo*

*In vivo* transplantation was carried out under general anaesthesia, mechanical ventilation and strict clinical standards. We used a porcine model of carotid artery replacement (end-to-end anastomosis using 7-0 prolene monofilament running suture by senior surgeons) with no immunosuppression. At surgery, small segments of the implanted D-hSV and the excised segment of carotid artery were retained and fixed in 10% formalin as pre-implant controls for histological comparisons. On completion of grafting, blood flow was confirmed up to 30min after reperfusion before wound closure. Antibiotic prophylaxis was used in line with clinical standards and the surgical wound was spread with approved veterinary antiseptic.

**Supplemental Material**

All the raw data used to generate this article is stored on a University of Bristol server with security access. We are happy to make the raw data available if necessary. We have extra aliquots of histology samples available if necessary. In-vivo pilot testing of the decellularised human vein was done at a certified MHRA GLP research facility for large animals at the University of Bristol and all the clinical research file/daily medical note for each experiment from surgery, critical care, and maintenance are and up to the termination procedures are stored under secure access in the facility and can be made available to the Editorial Office at any time if requested**.**

**References**

1. ISO 10993-5:2009; Biological evaluation of medical devices -- Part 5: Tests for in vitro cytotoxicity.

2. Zvarova B, Uhl FE, Uriarte JJ, et al. Residual Detergent Detection Method for Nondestructive Cytocompatibility Evaluation of Decellularized Whole Lung Scaffolds. *Tissue Eng Part C Methods* 2016; 22: 418-428. 2016/02/26. DOI: 10.1089/ten.TEC.2015.0439.

3. Mathapati S, Galla S, Sankaranarayanan K, et al. Qualitative and quantitative detection of sodium deoxycholic acid in decellularized tissue. *Indian J Thorac Cardiovasc Surg* 2010; 26: 129-131. DOI: 10.1007/s12055-010-0016-x.

**Tables**

Supplementary Table 1: Concentrations of SDS (% w/v and µM) used in experiment.

| [SDS] | % w/v | 0 | 7.5x10^-5^ | 1.0x10^-4^ | 2.5x10^-4^ | 5.0x10^-4^ | 7.5x10^-4^ | 1.0x10^-3^ | 2.5x10^-3^ | 5.0x10^-3^ | 7.5x10^-3^ | 1x10^-2^ | 1 |
| --- | --- | --- | --- | --- | --- | --- | --- | --- | --- | --- | --- | --- | --- |
|  | µM | 0 | 2.6 | 3.5 | 8.7 | 17.3 | 2.6x10^1^ | 3.5x10^1^ | 8.7x10^1^ | 1.7x10^2^ | 2.6x10^2^ | 3.5x10^2^ | 3.5x10^4^ |

Supplementary Table 2: Statistical test used for each experiment.

|  | Experiment | N= | Stats test |
| --- | --- | --- | --- |
| Small Vein Segments | Decellularisation Success: Nuclei count | 6 | Repeated measures one-way ANOVA with Dunnett’s multiple comparison |
|  | Decellularisation Success: DNA Content | 6 | Repeated measures one-way ANOVA with Dunnett’s multiple comparison |
|  | ECM Integrity | 6 | Repeated measures one-way ANOVA with Dunnett’s multiple comparison |
|  | SDS PCAEC Cytotoxicity:  Proliferation and Viability | 8 | Repeated measures one-way ANOVA with Dunnett’s multiple comparison |
|  | SDS PCASMC Cytotoxicity:  Proliferation and Viability | 6 | Repeated measures one-way ANOVA with Dunnett’s multiple comparison |
|  | Decellularised Organ Culture, BrdU | 5 | Paired t-test |
|  |  |  |  |
| Scale-Up (DhSV_FLOW_ vs. DhSV_ROLLER_) | Decellularisation Success: Nuclei count | 6 | One-way ANOVA with Tukey’s multiple comparison |
|  | Decellularisation Success: DNA Content | 10 | Repeated measures one-way ANOVA with Tukey’s multiple comparison |
|  | ECM Integrity | 7 | Repeated measures one-way ANOVA with Tukey’s multiple comparison |
|  | Residual SDS in washes | 8 | Two-way ANOVA with Tukey’s multiple comparison |
|  | Residual SDS in tissue | 5 | Paired t-test |
|  | Mechanical testing:  Burst Pressure and Compliance | 4-5 | One-way ANOVA with Tukey’s multiple comparison |
|  | Cell Seeding (ADSC, HUVEC, HSVSMC) | 4 | Unpaired t-test with Holm-Sidak multiple comparison |
